# CT Image-Based Biopsy to Aid Prediction of HOPX Expression Status and Prognosis for Non-Small Cell Lung Cancer Patients

**DOI:** 10.3390/cancers15082220

**Published:** 2023-04-10

**Authors:** Yu Jin, Hidetaka Arimura, YunHao Cui, Takumi Kodama, Shinichi Mizuno, Satoshi Ansai

**Affiliations:** 1 Division of Medical Quantum Science, Department of Health Sciences, Graduate School of Medical Sciences, Kyushu University, Fukuoka 812-8582, Japan; 2 Division of Medical Quantum Science, Department of Health Sciences, Faculty of Medical Sciences, Kyushu University, Fukuoka 812-8582, Japan; 3 Division of Medical Technology, Department of Health Sciences, Faculty of Medical Sciences, Kyushu University, Fukuoka 812-8582, Japan; 4 Laboratory of Genome Editing Breeding, Graduate School of Agriculture, Kyoto University, Kyoto 606-8520, Japan

**Keywords:** HOPX, CT image features, imaging biopsy, non-small cell lung cancer, radiogenomics

## Abstract

**Simple Summary:**

Recent studies have found that the HOPX gene functions as a tumor suppressor, and its expression status influences patients’ survival in NSCLC. However, the gene expression derived from the wet biopsy sampling has not shown the entire tumor microenvironment because NSCLC is a very heterogeneous disease. This study established an imaging biopsy with the radiogenomic signatures that links HOPX expression status and CT images to aid the prediction of HOPX expression status and the prognosis for lung cancer patients. Detecting gene expression status from CT images might be helpful to improve the accuracy of wet biopsy.

**Abstract:**

This study aimed to elucidate a computed tomography (CT) image-based biopsy with a radiogenomic signature to predict homeodomain-only protein homeobox (HOPX) gene expression status and prognosis in patients with non-small cell lung cancer (NSCLC). Patients were labeled as HOPX-negative or positive based on HOPX expression and were separated into training (*n* = 92) and testing (*n* = 24) datasets. In correlation analysis between genes and image features extracted by Pyradiomics for 116 patients, eight significant features associated with HOPX expression were selected as radiogenomic signature candidates from the 1218 image features. The final signature was constructed from eight candidates using the least absolute shrinkage and selection operator. An imaging biopsy model with radiogenomic signature was built by a stacking ensemble learning model to predict HOPX expression status and prognosis. The model exhibited predictive power for HOPX expression with an area under the receiver operating characteristic curve of 0.873 and prognostic power in Kaplan–Meier curves (*p* = 0.0066) in the test dataset. This study’s findings implied that the CT image-based biopsy with a radiogenomic signature could aid physicians in predicting HOPX expression status and prognosis in NSCLC.

## 1. Introduction

In the Global Cancer Statistics 2020, lung cancer was the second most commonly diagnosed cancer in the world, and it remained the leading cause of cancer death, with an estimated 1.8 million deaths last year [1]. Studies on gene expression analyses using ribonucleic acid (RNA) sequencing based on tumor biopsy sampling have presented new opportunities for the characterization of molecular signaling pathways in non-small cell lung cancer (NSCLC) that provides the basis for diagnosing and treating clinical lesions [2,3].

However, the gene expression derived from the biopsy rarely captures adequate information on the entire tumor environment because NSCLC is a disease with genetic and cellular heterogeneity [4]. In addition, it is often difficult to repeat the invasive biopsy in individual patients. The radiomics has the promising potential to transform medical images into quantitative information of the regions of interest (ROI) within tumors [5]. In other words, the tumor heterogeneity can be entirely captured with radiomics as imaging phenotypes by the three-dimensional imaging of tumors [6]. The image features extracted from the three-dimensional images could reflect heterogeneous tumor traits [7]. Moreover, the extraction of image features, i.e., image-based “biopsy” or “virtual biopsy”, can be performed repeatedly over time under an “as low as reasonably achievable” (ALARA) principle [8]. Radiogenomics is a rapidly growing research field that bridges image features and genomic information [9,10]. Radiogenomics, which is complementary or comparable to biopsies, could non-invasively identify spatially heterogeneous genomic information on tumors with increasing diagnostic accuracy [11,12].

Many previous studies have confirmed that images can reflect the heterogeneity of malignant tumors, and the radiogenomic analysis can provide heterogeneity measures for tumor characterization [13,14,15]. Nair et al. [13] reported that the epidermal growth factor receptor (EGFR) mutations in NSCLC could be predicted by radiogenomic models based on radiomic features extracted from 18F-Fluorodeoxyglucose (FDG) positron emission tomography/computed tomography (PET/CT) images. Kirienko et al. [14] also found that the combination of radiomic and genomic data could predict therapeutic outcomes in patients diagnosed with lung cancer using PET/CT. Although the majority of the radiogenomic analyses in lung cancer have been based on image features extracted from FDG PET/CT images, low-dose CT screening remains a recommended clinical service for lung cancer diagnosis under guidelines and reimbursement requirements in order to standardize the practice and optimize the balance between benefits and risks [16]. Ninomiya et al. [15] showed the potential of CT-based radiogenomics with topologically invariant features in the non-invasive identification of EGFR mutations in NSCLC patients. Therefore, this current study attempts to reveal the possibility of a radiogenomic analysis based on simple CT images to create an imaging biopsy to aid physicians in the prediction of the gene expression status and the progression of lung cancer. 

Moreover, many recent studies about radiogenomic investigations focused on EGFR mutation and achieved good predictions, which indicated the potential of linking genes to images [17,18]. Considering the complexity of the tumor microenvironment and the more precise treatment that should be applied to patients to achieve better survival, predictions of EGFR mutation status based on CT images can be extended to other genes [19,20]. Recently, researchers on RNA sequencing analysis have identified that the homeodomain-only protein homeobox (HOPX) gene may act as a tumor suppressor in NSCLC [21,22,23,24]. In lung cancer cell lines, HOPX is fully or partially methylated, which results in gene silencing or the down-regulation of its gene expression [21]. As a specific transcriptional regulator of differentiation, the low expression of HOPX may result in poorly differentiated tumors and a poor prognosis for patients diagnosed with lung cancer [22]. Therefore, predicting HOPX gene expression status before treatment will play a vital role in predicting the overall survival of patients diagnosed with lung cancer for the decision-making process regarding personalized treatment plans.

We hypothesized that HOPX expression status could be predicted using CT image-based radiogenomics or imaging biopsy, and a radiogenomic signature associated with HOPX expression could provide us with the prognostic power for stratification of patients with NSCLC into high- and low-risk groups. This study aimed to elucidate a radiogenomic signature based on CT images to predict the HOPX expression status and prognosis in patients with NSCLC.

## 2. Materials and Methods

### 2.1. Study Worflow

**Figure 1:**
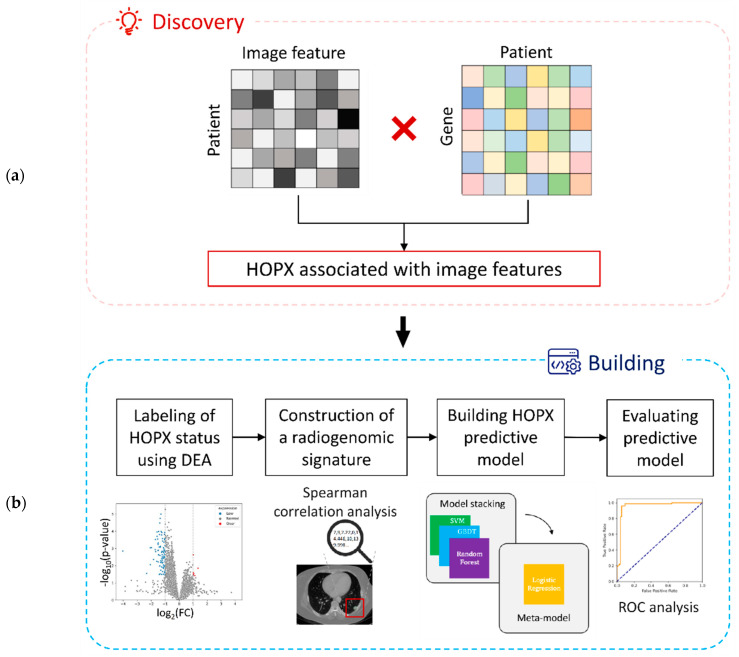
A workflow of this study: (**a**) a discovery step of image-associated genes and (**b**) a building step of an HOPX predictive model.

The study workflow is shown in Figure 1. The workflow consists of a discovery step of image-associated genes (HOPX) (Section 2.3 and Section 2.4) and a building step of an HOPX predictive model (Section 2.5, Section 2.6 and Section 2.7). 

In the discovery step of the image-associated gene, 1218 CT image features were extracted from a training dataset. Correlations between 10,674 gene expression data and 1218 image features derived from CT images were investigated. The HOPX gene has been found to be associated with image features in this study.

In the building step of the HOPX predictive model, all the patients were labeled as HOPX-negative and HOPX-positive using a differential gene expression analysis (DEA). Eight significant features associated with HOPX expression (absolute value of Spearman coefficients > 0.5) were selected for a radiogenomic signature from 1218 CT image features extracted from the training dataset. The predictive model of the imaging biopsy was built from a stacking ensemble learning to predict HOPX expression status and patient prognosis, and the model was evaluated with a receiver operating characteristic (ROC) analysis.

### 2.2. Clinical Cases

A total of 116 patients with both CT images with lung tumor contours and RNA sequencing data were selected from a dataset of NSCLC-radiogenomics in The Cancer Imaging Archive (TCIA), and those who did not have either CT images with contours or RNA sequencing data were excluded [25]. All the patients in this public dataset signed written informed consent forms according to the guideline of each institutional review board. Patient information is summarized in Table 1. The 116 patients were randomly split into training (*n* = 92, 79%) and test (*n* = 24, 21%) datasets so that there could be no statistically significant differences in age, gender, histology, stage, and HOPX status between the training and test datasets. The predictive model was developed from the training dataset, and it was evaluated for the test dataset. As these were retrospectively collected datasets from TCIA from two different centers (Stanford University Medical Center and Palo Alto Veterans Affairs Healthcare System, USA), the patients were scanned using different CT scanners with scanning parameters: pixel sizes of 0.59–0.98 mm (median: 0.78 mm), slice thicknesses of 0.625–2.5 mm (median: 1.25 mm), X-ray tube currents of 124–699 mA (mean: 220 mA), and X-ray tube voltages of 80–140 kV (mean: 120 kV). CT images of the patients were taken in the supine position with the arms at the side, from the lung apex to the adrenal glands, within a single breath-hold [25].

The original CT images with different pixel sizes and slice thicknesses were resampled using a linear interpolation method to produce three-dimensional (3D) CT images a with 1.0 × 1.0 × 1.0 mm^3^ isovoxel. The voxel size was determined by the median value of pixel size and slice thickness. The initial tumor regions were obtained as ROI without margins using an unpublished automatic segmentation algorithm used in a reference of 19 for tumor regions and viewed by two experienced radiologists to edit the disagreements in tumor boundaries as appropriate by using ePAD (electronic Physician Annotation Device) for the final ROI [25]. The ROI was also transformed into 3D ROI with a 1.0 × 1.0 × 1.0 mm^3^ isovoxel using the linear interpolation method.

Tumor tissue samples were collected from treatment-naïve subjects during surgical procedure. A 3 to 5 mm thick slice was cut along with the longest axis of the excised tissue, which was frozen within 30 min of excision. The RNA sequencing was performed on the tumor tissue samples, and then total RNA was converted into a library for paired-end sequencing on Illumina Hiseq according to the protocol for the Illumina TruSeq Sample preparation kit (Centrillion Biosciences, Palo Alto, CA, USA) [25].

### 2.3. Calculation of Image Features

A total of 1218 image features were extracted from the three types of images in this study: the original CT image, Laplacian of Gaussian (LoG), and wavelet filters. The LoG images enhanced the edges of ROIs. Wavelet-filtered images are the decomposition and approximation of the original image, which provides more detailed information about the texture. The image features consist of three groups: first-order (histogram) features (describing the statistical properties of voxel intensity histograms); shape features (measuring tumor dimensions and quantifying three-dimensional morphology); and texture features (describing joint probability and quantifying voxel correlations in gray level images), including gray level co-occurrence matrix (GLCM) features, gray level size zone matrix (GLSZM) features, gray level run length matrix (GLRLM) features, neighboring gray-tone difference matrix (NGTDM) features, and gray level dependence matrix (GLDM) features. Python 3.8.5 and Pyradiomics 3.0.1 were employed to extract image features from the ROIs [26]. All image features were normalized using z-score standardization to reduce basic differences in scale, range, and statistical distributions. 

### 2.4. Exploration of Significant Genes Associated with Image Features

The NSCLC-Radiogenomics database used in this study originally contained 60,660 genes for each patient. To ensure the accuracy of the calculations and avoid the introduction of noises that may affect results, genes with 80% missing values were excluded from the study, and the missing values in the remaining genes were replaced with zero. Finally, the gene expression matrix contained 10,674 genes for the next correlation analysis. To identify a significant gene, Spearman coefficients were computed for all combinations of 10,674 gene expression data and 1218 image features derived from CT images. The details of these methods are described in the Supplementary Materials. Finally, HOPX was found to be associated with CT image features.

### 2.5. Labeling of HOPX Status Using Differential Expression Analysis

All patients were labeled with HOPX status (positive or negative) using DEA based on RNA sequencing. A total of 116 cases were divided into HOPX-negative and HOPX-positive groups according to the log two-fold change (log_2_FC) in HOPX expression acquired from DEA. The HOPX-negative group was expected to have poorer survival than the HOPX-positive group. 

**Figure 2:**
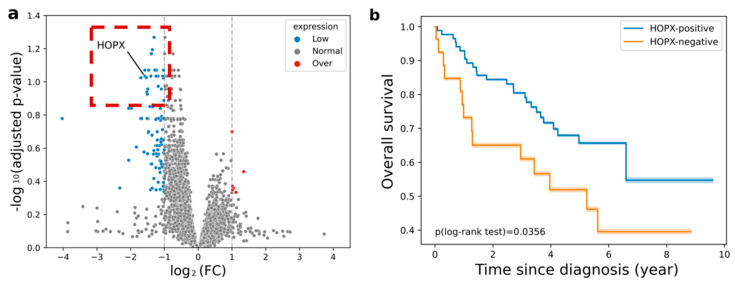
(**a**) Volcano plot for the relationship between differential expression (log_2_FC) and statistically significant difference of mean expressions (−log_10_(adjusted *p*-value)) obtained from short and long survival groups in terms of 10,674 genes including HOPX (log_2_FC = −1.575, *p*-value = 1.9 × 10^−4^, adjusted *p* = 0.092). The blue dot represents the low expression gene before correction, non-adjusted *p* < 0.05. The red dot represents the over-expression gene before correction, non-adjusted *p* < 0.05; (**b**) Kaplan–Meier curves of survival in the training dataset (*p* = 0.0356, log-rank test). Orange and blue curves represent HOPX-negative (poor survival) and HOPX-positive (good survival) groups, respectively.

In the DEA, patients were split into short-term and long-term survival groups based on the survival time (cutoff) of 2 years, which is the 75th percentile, upper quartile of the survival time [27]. FC for each patient is defined as the mean expression of the short survival group divided by that of the long survival one, which represents changes in each gene expression between the two groups [28]. The cutoff of log_2_FC would be set at −1 and 1 to find low- or over-expression genes within NSCLC patients. We employed the gene expression data, which were already normalized into fragments per kilobase of transcript per million mapped reads (FPKM) [25]. The *p*-value was calculated by Fisher’s exact test between the long survival and short survival group using the python package “SciPy (version 1.10.0)” after normalizing the data into negative binomial distribution [29]. The *p*-values in DEA were corrected by using the Benjamini–Hochberg (BH) method due to the multiple comparisons. However, since the *p*-values do not necessarily lead us to scientific conclusions [30,31], we utilized the survival analysis of KM curves (Figure 2b) and Spearman correlation analysis (Section 2.6), as well as DEA. The log_2_FC calculated from the DEA of HOPX will be set as the threshold to classify patients for the machine learning model. If log_2_FC < −1.57 (low expression of HOPX), the patient was considered HOPX-negative. Otherwise (normal and over-expression of HOPX), the patient was considered HOPX-positive.

A volcano plot for the relationship between differential expression (log_2_FC) and statistically significant difference of mean expressions (−log_10_(adjusted *p*-value)) obtained from the short and long survival groups in terms of 10,674 genes including HOPX is shown in Figure 2a. Previous studies using NSCLC cell lines (H2030, H2228, H157, H226, H2170, H1975, H23, A549, and H1299) [21] and NCI Director’s Challenge Cohort (DCC; *n* = 442) [24] reported that patients with low HOPX expression were more likely to exhibit poor overall survival. The prognostic power of the HOPX expression status was verified in the training dataset of this study. As shown in Figure 2b, the survival rates of the two groups were statistically significantly different. 

### 2.6. Construction of the Radiogenomic Signature

Eight significant features that correlated with HOPX expression were selected for the radiogenomic signature from the CT image features extracted from the training dataset. Image features with higher correlations with the expression status of HOPX were selected by a Spearman correlation analysis (the absolute value of Spearman coefficients > 0.5) as radiogenomic signature candidates. The *p*-values in the correlation analysis were corrected by the BH correction method. Among those image features, the least absolute shrinkage and selection operator (LASSO) regression model selected the best combination of features as the final radiogenomic signature for CT-image based biopsy.

### 2.7. Building of the Imaging Biopsy Model

**Figure 3:**
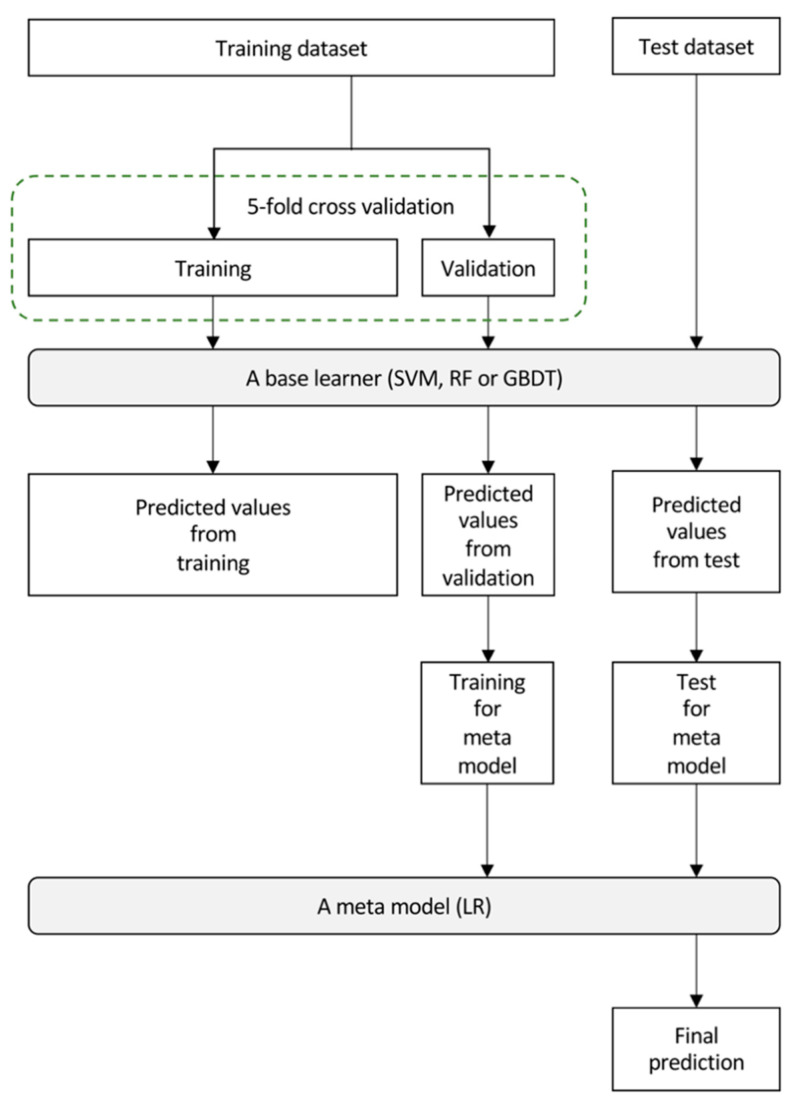
A workflow for building of an imaging biopsy model based on stacking ensemble learning for this study.

Figure 3 depicts the workflow for building an imaging biopsy model based on stacking ensemble learning for this study. A stacking ensemble machine learning model was built to predict the expression status of HOPX. The stacking ensemble machine learning model used in this study (building predictive model) consists of two levels of learning steps, i.e., the construction of base learners and the building of a meta-learner to learn all predictions from the base models as input features [32]. A support vector machine (SVM), random forest (RF), and gradient boosting decision tree (GBDT) were constructed as the base learners in the first level, and the predicted values from these models were assembled as new input variables for the meta-model in the second level. From the original training dataset, 20% of the cases were excluded as a validation dataset for training the meta-model at the second level in stacking. The predicted values represent the probability of classification as HOPX-negative or HOPX-positive. The adaptive synthetic sampling approach (ADASYN) and synthetic minority oversampling technique (SMOTE) were used as the data augmentation method for the stacking model to learn from imbalanced datasets to achieve better classification results [33,34]. The Bayesian optimization searched for the best hyperparameters of SVM, RF, and GBDT [35]. During a 5-fold cross-validation test, predicted values by the base models for all of our training data were produced to increase the training data to the meta-model. A logistic regression (LR) model was built as the meta-model (the second level) to combine the predicted values from the base learners and produce the final predicted value. The threshold value for the predicted value in the meta-model was set to 0.5. A patient with a final predicted value greater than 0.5 is considered HOPX-negative. The patient less than 0.5 is regarded as HOPX-positive. 

### 2.8. Evaluation of the Imaging Biopsy Model

The area under the ROC curve (AUC), accuracy, sensitivity, and specificity in the ROC analysis were evaluated for the prediction of HOPX status using the proposed approach. The prognostic power of the imaging biopsy model with different radiogenomic signatures was evaluated using *p*-values in log-rank tests between Kaplan and Meier curves for the two groups classified by the model. *P*-values lower than 0.05 were considered statistically significant in this study.

## 3. Results

### 3.1. Radiogenomic Features

**Figure 4:**
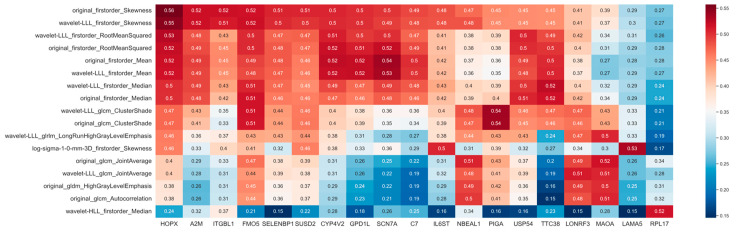
Correlation coefficient (absolute value) heatmap between gene expression (HOPX) and image features. Red color refers to high correlations.

Spearman coefficients for all combinations of 10,674 gene expression data and 1218 CT image features were calculated to identify image-feature-related genes. A heatmap of relatively higher Spearman coefficients between 19 gene expressions (HOPX, A2M, ITGBL1, FMO5, SELENBP1, SUSD2, CYP4V2, GPD1L, SCN7A, C7, IL6ST, NBEAL1, PIGA, USP54, TTC38, LONRF3, MAOA, LAMA5 and RPL17) and seventeen image features is shown in Figure 4. Among all these genes, only the HOPX gene indicated the absolute value of Spearman coefficients higher than 0.5 for most image features, eight features, and was also detected in differential expression analysis with a low expression level. Both the *p*-value and adjusted *p*-value of HOPX in Spearman correlation analysis were lower than 0.05.

### 3.2. Prediction Power of HOPX Expression Status and Prognosis

The performances of the imaging biopsy models with four radiogenomic signature candidates are shown in Table 2, including AUC, accuracy, sensitivity, and specificity for both the training and test datasets. The different numbers of features in the signatures were obtained by changing a regularization parameter of LASSO. The best predictive model was the model with imaging biopsy D, consisting of two radiogenomic features, which showed an AUC of 0.965 for the training dataset and an AUC of 0.873 for the test dataset.

**Figure 5:**
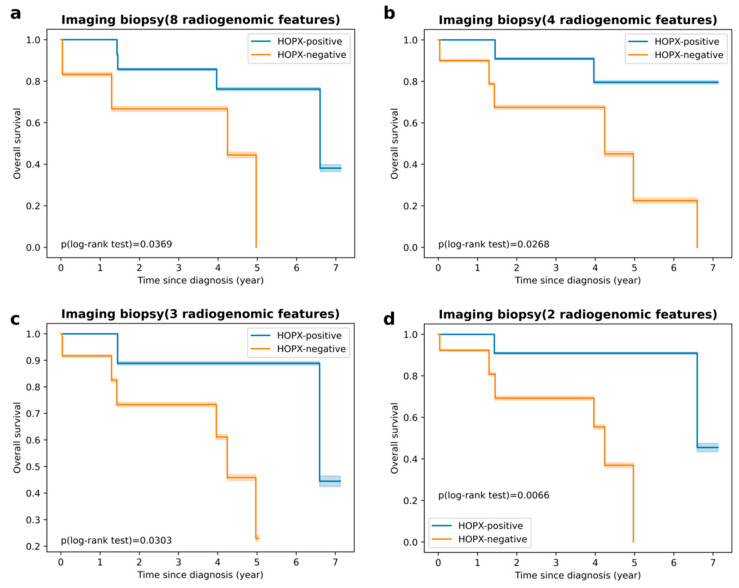
Kaplan–Meier curves of survival in test dataset: (**a**) survival curves of imaging biopsy consisting of 8 radiogenomic features (*p* = 0.0369, log-rank test); (**b**) survival curves of imaging biopsy consisting of 4 radiogenomic features (*p* = 0.0268, log-rank test); (**c**) survival curves of imaging biopsy consisting of 3 radiogenomic features (*p* = 0.0303, log-rank test); (**d**) survival curves of imaging biopsy consisting of 2 radiogenomic features (*p* = 0.0066, log-rank test).

Figure 5 shows the Kaplan–Meier survival curves predicted by the four imaging biopsies for the test dataset. Orange and blue curves represent the predicted HOPX-negative (poor survival) and HOPX-positive (good survival) groups, respectively. Imaging biopsy D was selected as the final radiogenomic signature, which showed the most statistically significant difference (*p* = 0.0066). The predictive model using imaging biopsy D has prognostic power for HOPX expression status and overall survival.

### 3.3. Final Image Biopsy with Radiogenomic Signature

**Figure 6:**
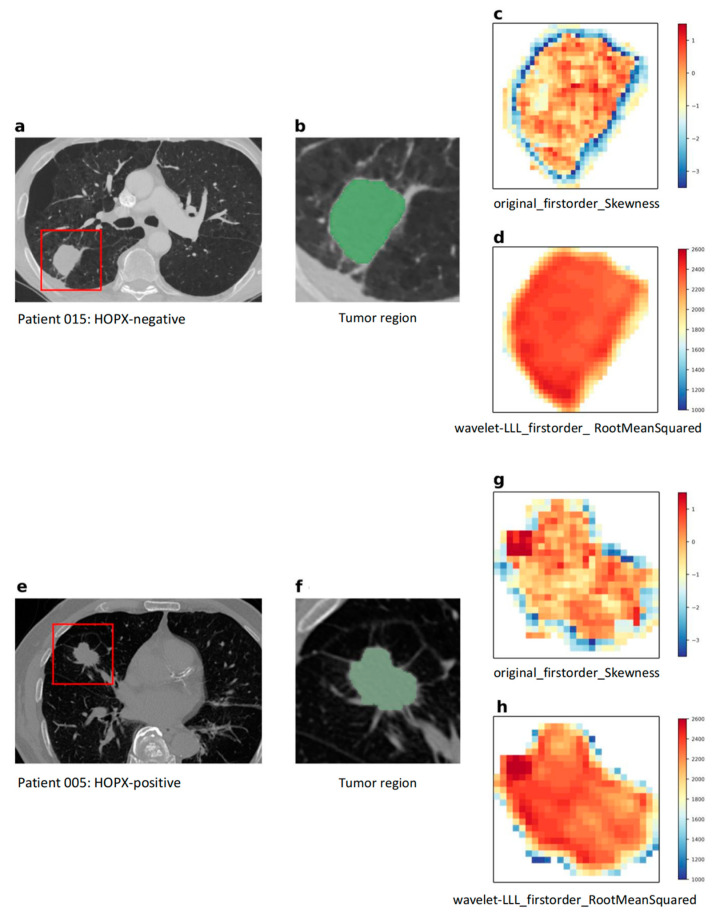
Image feature maps of “original_firstorder_Skewness” and “wavelet-LLL_firstorder_RootMeanSquared,” for two patients with different HOPX expression statuses: (**a**) a CT image from an HOPX-negative patient; (**b**) a tumor region (ROI) for the HOPX-negative patient; (**c**,**g**) maps of “original_firstorder_Skewness” image feature extracted from ROI; (**d**,**h**) maps of “wavelet-LLL_RootMeanSquared” image feature extracted from ROI; (**e**) a CT image from an HOPX-positive patient; (**f**) a tumor region (ROI) for the HOPX-positive patient.

**Figure 7:**
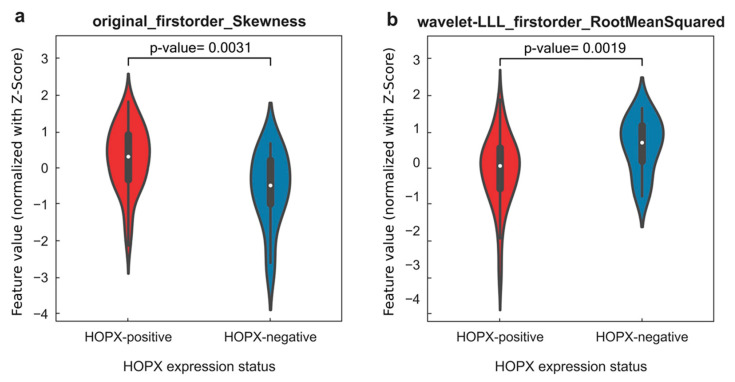
(**a**) Violin plot of image feature “original_firstorder_Skewness” of all HOPX-positive and HOPX-negative patients, which showed a significant difference in data distribution (*p* = 0.0031); (**b**) violin plot of image feature “wavelet-LLL_firstorder_RootMeanSquared” of all HOPX-positive and HOPX-negative patients, which showed a statistically significant difference in data distribution (*p* = 0.0019).

Figure 6 shows image feature maps of “original_firstorder_Skewness” (Spearman coefficient = 0.557, *p* = 8.05 × 10^−9^, adjusted *p* = 9.50 × 10^−6^) and “wavelet-LLL_firstorder_RootMeanSquared” (Spearman coefficient = 0.527, *p* = 6.69 × 10^−8^, adjusted *p* = 2.83 × 10^−5^), which are two significant image features in the final radiogenomic signature, for two patients with different HOPX expression statuses. This figure is discussed in Discussion Section. The means of “original_firstorder_Skewness” and “wavelet-LLL_firstorder_RootMeanSquared” also showed statistically significant differences between HOPX-positive and HOPX-negative patients, as shown in the violin plots in Figure 7.

## 4. Discussion

This study found that the HOPX gene out of 10,674 genes was associated with CT image features. The machine learning model built by different imaging biopsies based on radiogenomic features for the HOPX gene showed an ability to predict patients’ lung cancer prognosis, which suggested that the gene expression and image features can be integrated to create an image-based biopsy to aid the prediction of the prognosis of cancers. The imaging biopsy consisted of eight radiogenomic features (first-order features), which describe the heterogeneity of voxel intensities within the tumors on CT images. We hypothesized that the reason for this phenomenon was related to the biological functions of HOPX in the lungs and lung cancer. 

The first-order feature of skewness shown in Figure 6c,g measured the asymmetry of the histograms of the voxel values concerning the mean value. The tumor regions near the boundaries for the HOPX-negative patient (c) showed higher skewness pixel values than those for the HOPX-positive patient (g), which may suggest more aggressive cell invasion and less differentiation in the HOPX-negative patient. The data distribution of the first-order feature of skewness between HOPX-negative and HOPX-positive patients in Figure 7a also showed a similar phenomenon: HOPX-negative patients showed a more discrete data distribution. The root mean square (RMS) shown in Figure 6d,h is the square root of the mean of all squared pixel values, which is another measure of the magnitude of the pixel values. The intratumor regions of the HOPX-negative patient showed higher RMS pixel values than those for the HOPX-positive patients. These results are two examples of the ones shown in Figure 7b, where the RMSs for the HOPX-negative patients were relatively higher than those for the HOPX-positive patients. These results suggest that the heterogeneity in the HOPX-negative patients was greater than that in the HOPX-positive patients. 

In recent years, several studies have attempted to elucidate the tumor-promoting and tumor-suppressing pathways that cause NSCLC in order to achieve more effective treatment. A particularly crucial role in the modulation of heart and lung development is played by HOPX [31]. By tracing the signaling pathway of HOPX, researchers discovered that it has a significant influence on inhibiting lung cancer cell proliferation and suppressing tumor cell migration and invasion, which results in a lower expression in NSCLC and a poor prognosis for patients [21]. HOPX is also involved in part of a transcriptional program that is related to distal airway epithelial differentiation and lung adenocarcinoma (LUAD) progression, in which HOPX is considered to induce cellular senescence with the activation of Ras/MAPK signaling and inhibition of the Akt pathway [24,36]. It is one of the important nodes in a lineage-selective pathway (GATA6, HOPX, and NKX2-1) that directly connects effectors of airway epithelial specification to the inhibition of metastasis in the LUAD subtype [24].

Recent studies have found that alveolar type 2 (AT2) cells may be the cells of origin of human LUAD, which cause dedifferentiation to produce a stem cell-like state that initiates and retains cancer progression [37,38]. The low expression of HOPX may exacerbate the process of alveolar cell dedifferentiation into distal airway stem cells, leading to poor survival [24]. There may be some relationships between cell differentiation and the phenotype of first-order features, which could be seen from the selected image features for establishing the radiogenomic signature in our study. 

Genes associated with image features, i.e., SELENBP1 and SUSD2, other than HOPX were found in both Spearman correlation analysis and DEA. SELENBP1 (Selenium-binding protein 1) was a gene of an important tumor suppressor during the origin and development of NSCLC and may be a novel targeted biomarker [39]. SUSD2 (Sushi Domain Containing 2) was indicated to serve as a new prognostic and potential therapeutic target in lung adenocarcinoma [40].

Our study had three limitations. Firstly, the CT images used for this study were acquired from different CT scanners with various pixel sizes (0.59–0.98 mm), slice thicknesses (0.625–2.5 mm), X-ray tube currents (124–699 mA), and X-ray tube voltages (80−140 kV). The original CT images were resampled using a linear interpolation method to produce isotropic CT images with 1.0 × 1.0 × 1.0 mm^3^. Since small pixel sizes and thicknesses were differently used in the CT scanning, those images could include different noise, which may affect the results in this radiogenomic study. To reduce the effects induced by differences in acquisition protocol and scanner, harmonization techniques [41] such as the ComBat algorithm should be applied to the further studies.

Secondly, the performance of the ensemble machine learning model with imaging biopsy consisting of eight and three radiogenomic features produced a low specificity (Table 2) for HOPX-positive patients in the test dataset, which may indicate that the model achieved an overfitting result. There could be two possible reasons for these results. The first reason was that the patient cohort for this study was obtained from TCIA, which was collected several years ago and included a relatively small number of cases. This may have limited the ability of the model to predict the gene expression status, and it also caused imbalanced data for gene expression status that would make training more complicated. Further evaluation using a larger number of databases, including different institutions and image quality, is necessary for a subsequent study. The second reason is the choice of the machine learning model. A more appropriate machine learning model should be used to reduce overfitting in the training dataset and improve the specificity of the test dataset. For example, XGboost, which is an improvement of GBDT and its loss function, may reduce overfitting [42]. 

The third limitation may be that we employed only histogram and texture image features associated with intratumor intensity heterogeneity, which could lead to low specificity. Therefore, various types of image features, such as shape features [26] with peritumoral regions that may represent infiltrative cancer cells, should be considered in future studies.

## 5. Conclusions

In conclusion, HOPX, which regulates tumor-suppressive functions in lung cancer, was found to be associated with image features in this study. This study elucidated an imaging biopsy with the radiogenomic signature based on CT images to aid the prediction of the HOPX expression status and prognosis in patients with NSCLC. Our study on radiogenomic signatures revealed the potential to assist in predicting lung cancer prognosis and demonstrated the possibility of linking imaging data to genetic information at the molecular level. Detecting gene expression status from CT images might improve the accuracy of wet biopsies.

## Figures and Tables

**Table 1:** Patients’ information.

		All	Training Dataset	Test Dataset	*p*-Value (Method)
Number of Cases		(*n* = 116)	(*n* = 92)	(*n* = 24)	
Age	<60	17	12 (70.59%)	5 (29.41%)	0.976
	≥60	99	80 (80.81)	19 (19.19%)	(Mann-Whitney)
Gender	Male	87	67 (77.01%)	20 (22.99%)	0.427
	Female	29	25 (86.21%)	4 (13.79%)	(Chi-squared)
Histology	LUAD ^1^	88	70 (79.55%)	18 (24.45%)	0.621
	LUSC ^2^	25	19 (76.00%)	6 (24.00%)	(Chi-squared)
	NOS ^3^	3	3 (100.00%)	0 (0.00%)	
Stage	0	5	4 (80.00%)	1 (20.00%)	0.386
	Ia	44	38 (86.36%)	6 (13.64%)	(Chi-squared)
	Ib	27	21 (77.78%)	6 (22.22%)	
	IIa	11	7 (63.64%)	4 (36.36%)	
	IIb	9	5 (55.56%)	4 (44.44%)	
	IIIa	15	12 (80.00%)	3 (20.00%)	
	IIIb	1	1 (100.00%)	0 (0.00%)	
	IV	4	4 (100.00%)	0 (0.00%)	
HOPX status	HOPX-negative	27	20 (74.07%)	7 (25.93%)	0.620
	HOPX-positive	89	72 (80.90%)	17 (19.10%)	(Chi-squared)

NOTE: The ratios of HOPX-negative patients to all patients in the training and test datasets were 21.74% and 29.17%, respectively, and those of HOPX-positive patients were 78.26% and 70.83%, respectively. ^1^ LUAD: lung adenocarcinoma; ^2^ LUSC: lung squamous cell carcinoma; ^3^ NOS: not otherwise specified.

**Table 2:** Performances of the ensemble learning model—Stacking.

	Training Dataset				Test Dataset			
	AUC	Accuracy	Specificity	Sensitivity	AUC	Accuracy	Specificity	Sensitivity
**Imaging biopsy A, consisting of** **8 radiogenomic features:** original_firstorder_Skewness original_firstorder_Median original_firstorder_Mean original_firstorder_RootMeanSquared wavelet-LLL_firstorder_Skewness wavelet-LLL_firstorder_Median wavelet-LLL_firstorder_Mean wavelet-LLL_firstorder_RootMeanSquared	0.995	0.939	0.985	0.920	0.664	0.625	0.286	0.764
**Imaging biopsy B, consisting of** **4 radiogenomic features:** original_firstorder_Skewness original_firstorder_Mean wavelet-LLL_firstorder_Median wavelet-LLL_firstorder_RootMeanSquared	0.998	0.986	0.705	0.714	0.672	0.708	0.706	0.714
**Imaging biopsy C, consisting of** **3 radiogenomic features:** original_firstorder_Skewness wavelet-LLL_firstorder_Median wavelet-LLL_firstorder_RootMeanSquared	0.953	0.890	0.904	0.706	0.706	0.625	0.588	0.714
**Imaging biopsy D, consisting of** **2 radiogenomic features:** original_firstorder_Skewness wavelet-LLL_firstorder_RootMeanSquared	0.965	0.876	0.877	0.877	0.873	0.750	0.647	1.000

## Data Availability

The Python source codes of this study are available as part of the replication package at [https://github.com/ginyuu23/Radiogenomics-analysis.git]. The CT image and gene information of lung cancer patients were from the public database, TCIA.

## Supplementary Materials

The following supporting information can be downloaded at: https://www.mdpi.com/article/10.3390/cancers15082220/s1, Figure S1: Calculation procedure of Spearman coefficients to find the significant gene; Figure S2: The data distribution analysis in a Quantile-Quantile plot with the *p*-value (chi-squared test) of the final radiogenomic signature, consisting of two image features (“original_firstorder_Skewness” and “wavelet-LLL_firstorder_RootMeanSquared”), Figure S3: The entire heatmap for Spearman correlation analysis; Table S1: Case IDs of patients used in the training dataset; Table S2: Case IDs of patients used in the test dataset.
