# Supplementary material for: CT Image-Based Biopsy to Aid Prediction of HOPX Expression Status and Prognosis for Non-Small Cell Lung Cancer Patients
Source: Cancers (Basel). 2023 Apr 10;15(8):2220. doi: 10.3390/cancers15082220 (PMC10136849; doi:10.3390/cancers15082220)

Supplementary Materials

Table S1 Case IDs of patients used in the training dataset

Training dataset (n=92), Case IDs							
R01-004	R01-027	R01-048	R01-064	R01-080	R01-104	R01-120	R01-136
R01-006	R01-028	R01-049	R01-065	R01-083	R01-105	R01-122	R01-137
R01-007	R01-029	R01-051	R01-066	R01-084	R01-107	R01-123	R01-138
R01-012	R01-031	R01-052	R01-067	R01-091	R01-108	R01-124	R01-139
R01-013	R01-032	R01-054	R01-068	R01-093	R01-110	R01-126	R01-140
R01-014	R01-037	R01-055	R01-069	R01-094	R01-111	R01-127	R01-141
R01-016	R01-039	R01-057	R01-072	R01-097	R01-112	R01-128	R01-144
R01-017	R01-040	R01-059	R01-073	R01-098	R01-113	R01-129	R01-146
R01-018	R01-041	R01-060	R01-076	R01-099	R01-115	R01-131	
R01-021	R01-042	R01-061	R01-077	R01-100	R01-116	R01-132	
R01-022	R01-043	R01-062	R01-078	R01-101	R01-117	R01-133	
R01-023	R01-046	R01-063	R01-079	R01-103	R01-118	R01-134	

Table S2 Case IDs of patients used in the test dataset

Test dataset (n=24), Case IDs							
R01-003	R01-024	R01-034	R01-056	R01-089	R01-106	R01-121	R01-135
R01-005	R01-026	R01-035	R01-071	R01-096	R01-109	R01-125	R01-142
R01-015	R01-033	R01-038	R01-081	R01-102	R01-114	R01-130	R01-145

Figure S1 Calculation procedure of spearman coefficients to find the significant gene. The data distribution was analyzed by a quantile-quantile plot and chi-squared test. Spearman coefficients were computed for all combinations between 10674 gene expression data and 1218 image features derived from computed tomography (CT) in the training dataset (n=92). The combinations of image feature and gene with spearman coefficients greater than 0.5 were considered to be correlated with each other. Among all selected genes, HOPX gene showed highest coefficients with all selected image features, which is the significant gene studied in this paper.

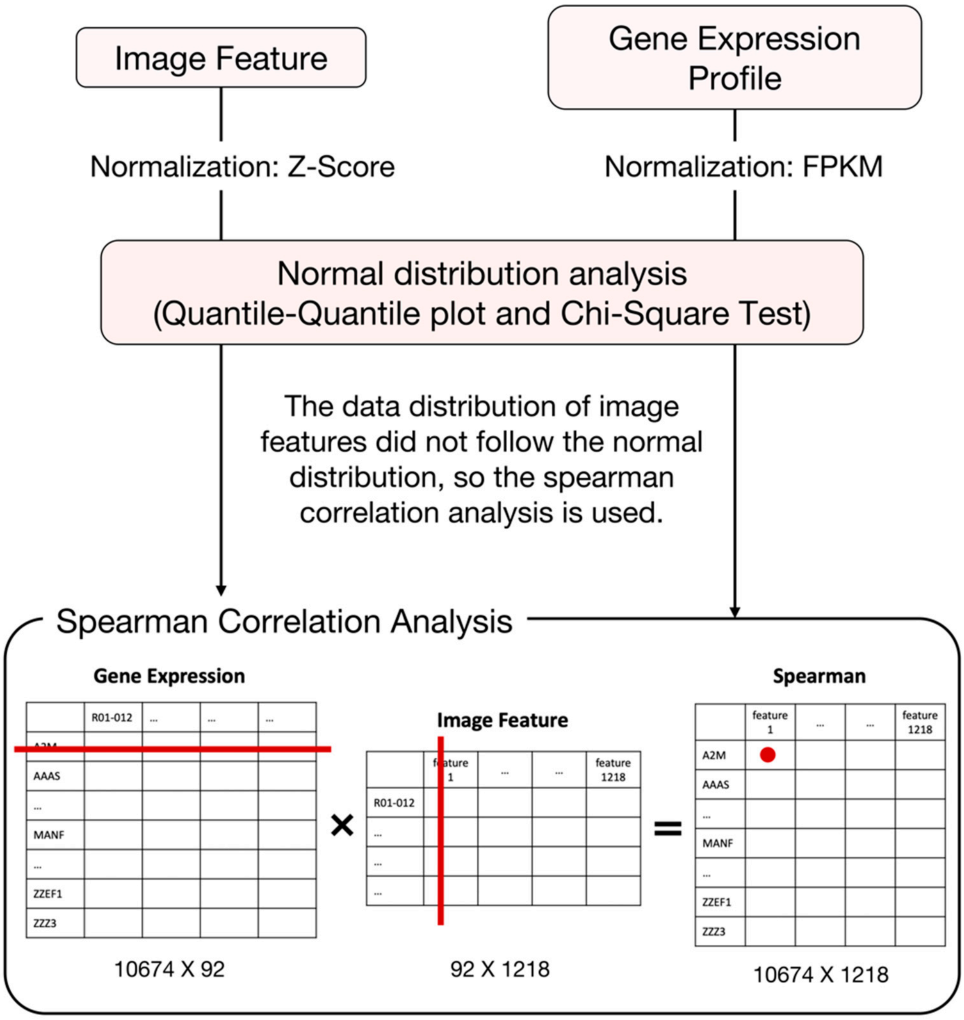

Figure S2 The data distribution analysis in a Quantile-Quantile plot with the p-value (Shapiro-Wilks test) of the final radiogenomic signature, consisting of two image features (“original_firstorder_Skewness” and “wavelet-LLL_firstorder_RootMeanSquared”). The data distribution of these two image features showed significant differences between the normal distribution (p=0.032 and p=0.003, respectively).

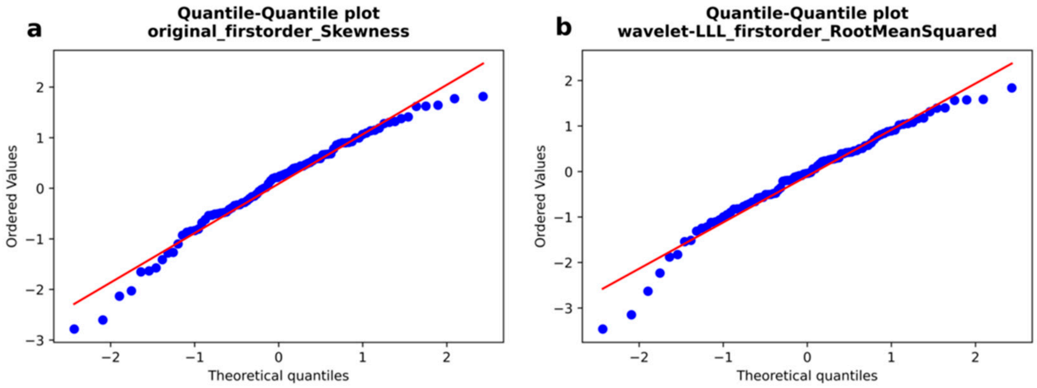

FigureS3 The entire heatmap for Spearman correlation analysis

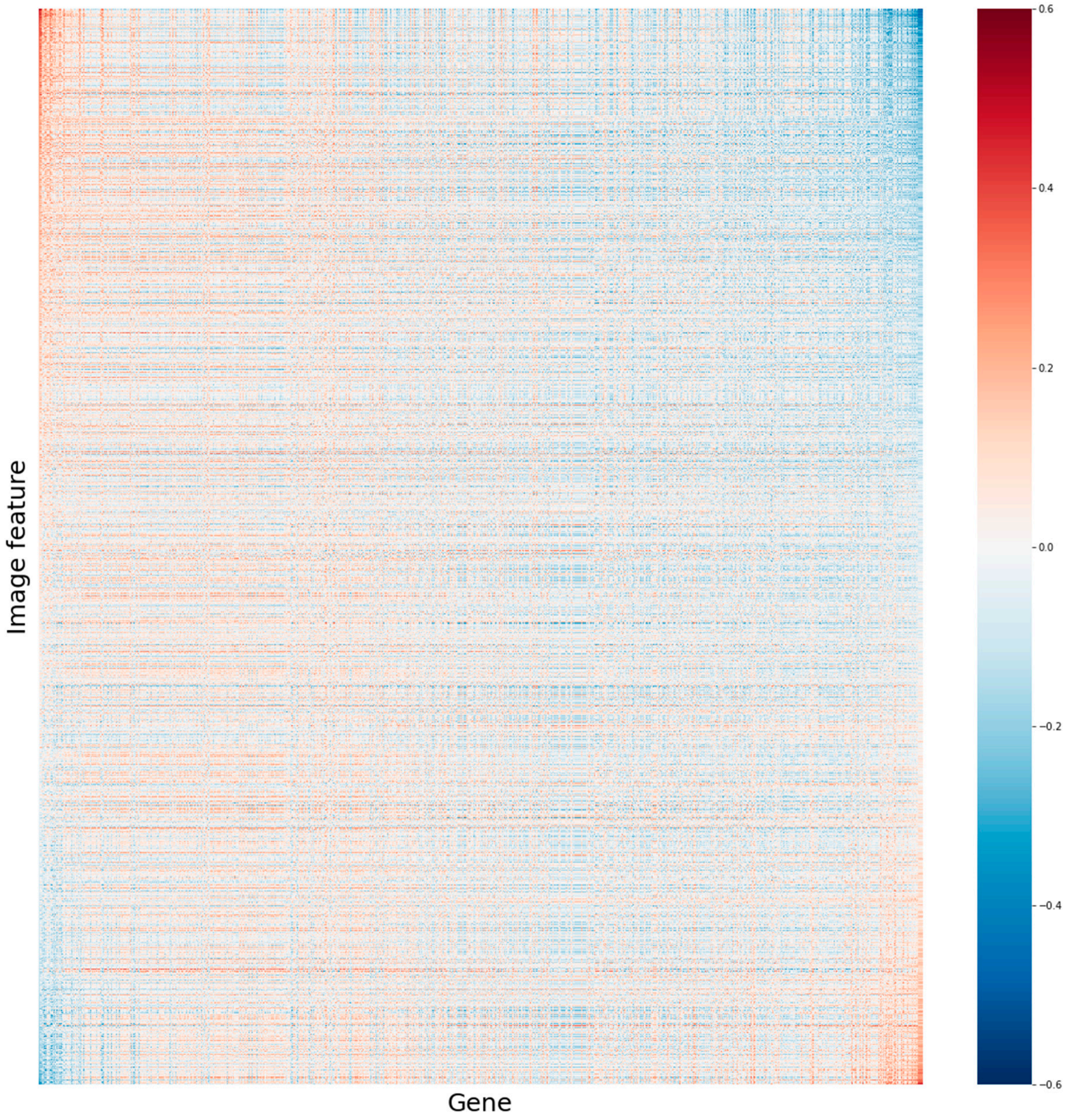

Supplement: Supplementary file 1 [file cancers-15-02220-s001.zip › cancers-2233186-supplementary.pdf]
